# Lactylation-driven therapeutic resistance in cancer: Mechanisms and therapeutic opportunities

**DOI:** 10.1016/j.gendis.2025.101935

**Published:** 2025-11-17

**Authors:** Wanghao Zhang, Guanglong Huang, Woheng Tang, Jiaxian Li, Jingxian Chen, Yaojiang Feng, Kaichen Li, Can Pan, Shunshen Li, Huayang Zhang, Rongxu Ye, Hao Long, Guo-zhong Yi

**Affiliations:** a Department of Neurosurgery, Nanfang Hospital, Southern Medical University, Guangzhou, Guangdong 510515, China; b The Laboratory for Precision Neurosurgery, Nanfang Hospital, Southern Medical University, Guangzhou, Guangdong 510515, China; c The Second Clinical Medical College, Southern Medical University, Guangzhou, Guangdong 510260, China; d The First Clinical Medical College, Southern Medical University, Guangzhou, Guangdong 510515, China; e School of Public Health, Southern Medical University, Guangzhou, Guangdong 510515, China; f Nanfang Glioma Center, Guangzhou, Guangdong 510515, China; g Institute of Brain Disease, Nanfang Hospital, Southern Medical University, Guangzhou, Guangdong 510515, China

**Keywords:** Lactate, Lactylation, Therapeutic resistance, Tumor microenvironment, Warburg effect

## Abstract

Lactylation, a type of post-translational modification (PTM) of proteins driven by cancer metabolic reprogramming, not only offers new perspectives on the Warburg effect but also has drawn increasing attention due to its critical roles in tumorigenesis and therapeutic resistance. Given its significant potential for precision cancer therapy, this review first integrates recent advancements in lactylation research by systematically summarizing newly identified lactylation writers, readers, and erasers, as well as their involvement in feedback loops and crosstalk with other modifications. Subsequently, we elaborate on how histone and non-histone lactylation contribute to both intrinsic and acquired resistance to radiotherapy, chemotherapy, targeted therapy, and immunotherapy. Key mechanisms encompass maintaining cancer stemness, enhancing DNA damage repair, reprogramming metabolic pathways, inhibiting ferroptosis, and promoting an immunosuppressive tumor microenvironment. Finally, we evaluate preclinical strategies targeting lactylation, including inhibition of lactate metabolic pathways and direct modulation of lactylation-modifying enzymes or lactylated proteins, while critically assessing mechanistic challenges and early-phase clinical trial outcomes. Our analysis establishes a theoretical framework and actionable roadmap for the development of lactylation-based precision therapies in oncology.

## Introduction

Cancer remains one of the most pressing global public health challenges. The rising burden of cancer, driven by environmental pollution, unhealthy lifestyle choices, increasing social stress, and population aging,^1^ persists despite significant advances in cancer treatment. While modern oncology has developed comprehensive therapeutic strategies, including surgery, radiotherapy, chemotherapy, targeted therapy, and immunotherapy, most patients eventually experience disease progression due to treatment resistance.

This therapeutic challenge originates from two distinct mechanisms: intrinsic resistance present before treatment in some tumors, and acquired resistance that develops during therapy in initially responsive cancers. Researchers have identified multiple mechanisms underlying tumor therapeutic resistance, including but not limited to drug target loss, up-regulation of drug efflux transporters,^2^^,^^3^ activation of DNA damage repair pathways,^4^ evasion of apoptosis,^5^ and dynamic cellular phenotype switching.^6^ Importantly, growing evidence highlights the crucial role of non-genetic mechanisms, particularly epigenetic regulation, metabolic reprogramming, and post-translational modifications (PTMs), in dynamically regulating these resistance pathways through rapid adaptation to cellular environmental changes.

Among these mechanisms, protein lactylation stands out as particularly noteworthy. This PTM uniquely employs lactate, a key metabolic byproduct in the tumor microenvironment, as its substrate. By modifying proteins and affecting gene transcription, lactylation serves as a critical link between metabolic reprogramming and epigenetic regulation. Most significantly, emerging evidence strongly connects lactylation with both intrinsic and therapy-induced acquired resistance during tumor development and progression. This review first summarizes recent advances in understanding lactylation pathways, and then analyzes how lactylation confers cellular resistance properties. Finally, we evaluate therapeutic strategies targeting lactylation to overcome treatment resistance, providing both theoretical foundations and translational insights for developing novel precision cancer therapies.

## Overview of lactylation

### The discovery of lactylation

The groundbreaking observation by Professor Otto Warburg over a century ago—that cancer cells preferentially convert glucose to lactate via glycolysis even under aerobic conditions (the Warburg effect)^7^—sparked numerous hypotheses regarding its underlying mechanisms. These included Warburg’s own mitochondrial dysfunction theory of tumorigenesis,^8^ as well as alternative explanations such as the faster ATP production rate of glycolysis than of oxidative phosphorylation, the greater demand for biosynthetic precursors than energy in proliferating cells, and the redox imbalance hypothesis. These discoveries fundamentally challenge the traditional view of lactate as merely a metabolic waste product.

In 2019, Professor Yingming Zhao’s team made a paradigm-shifting discovery that further illuminated lactate’s critical role in tumor biology. Using high-performance liquid chromatography–tandem mass spectrometry (HPLC-MS/MS), they identified lactate as a novel modification donor that drives histone lysine lactylation.^9^ Subsequent studies have revealed extensive lactylation sites on both histone and non-histone proteins.

To date, three distinct lysine lactylation isoforms have been identified: (1) lysine L-lactylation (K_L-la_), (2) lysine D-lactylation (K_D-la_), and (3) N-ε-(carboxyethyl) lysine (K_ce_). K_D-la_ and K_ce_ are primarily generated through non-enzymatic modifications, driven by S-d-lactoylglutathione (D-LGSH) and methylglyoxal (MGO), respectively.^10^ Their abundance is relatively low in human cells and is mainly detected under conditions where the glyoxalase system is impaired. In contrast, K_L-la_ is the predominant form in tumor cells, with its level closely linked to the glycolytic activity of cancer cells and uniquely responsive to hypoxia,^11^, ^12^, ^13^ suggesting its strong association with the malignant phenotypes of tumor cells. Given these differences, this review specifically focuses on l-lactate (Lac or la) and lysine L-lactylation (Kla). Unless otherwise specified, all references to “lactate” and “lactylation” in this review pertain to these forms.

### Enzymes involved in lactylation

**Figure 1:**
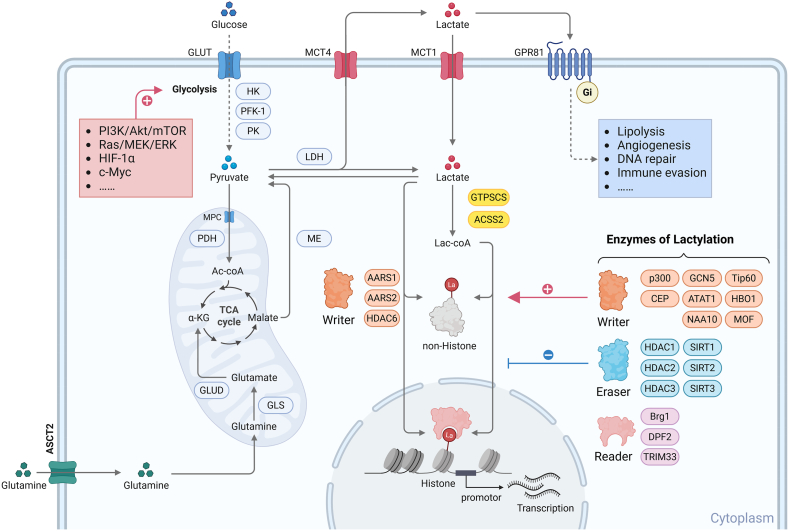
Overview of l-lactate metabolism and L-lactylation. Tumor cells generate lactate through endogenous glycolytic metabolism, glutaminolysis, and exogenous uptake. Beyond its role as a metabolic intermediate that enters the tricarboxylic acid (TCA) cycle for energy production, lactate also functions as a signaling molecule via autocrine or paracrine mechanisms to activate downstream signaling pathways of GPR81. More importantly, lactate serves as a donor for post-translational modifications (PTMs), driving lactylation modifications. Based on substrate specificity, lactylation can be categorized into histone lactylation and non-histone lactylation. The regulation of lactylation modifications requires the coordinated action of multiple enzymes, including lactyl-CoA synthetases, writer enzymes (some of which are lactyl-CoA-independent), eraser enzymes, and reader enzymes. La lactate. Created with BioRender.com.

In mammalian systems, L-lactylation modifications require specific “writer” enzymes. AARS1/2^14−16^ and HDAC6^17^ can directly utilize ATP to bind lactate, catalyzing the formation of lactyl-AMP, which is then transferred to lysine acceptor residues on target proteins. Other identified lactylation writer enzymes depend on lactyl-CoA to attach the modification to lysine residues through enzymatic reactions, and these belong to four major acyltransferase families of HATs: the p300/CBP family including p300^9^ and CBP; the MYST family containing Tip60 (KAT5),^18^ HBO1 (KAT7),^19^ and MOF (KAT8)^20^; the GNAT family with GCN5 (KAT2A)^21^; as well as ATAT1^22^ and NAA10.^23^ Currently, only two lactyl-CoA synthetases have been identified: ACSS2^24^ and GTPSCS.^25^ The removal of lactylation modifications relies on “eraser” enzymes with deacylase activity, such as HDAC1-3 and SIRT1-3.^26^ Interestingly, although most enzymes exhibit functional redundancy, some have been found to function in an environment-dependent manner with target protein specificity. For instance, in neurons, the primary “writer” enzyme HDAC6 can catalyze the lactylation of α-tubulin within a lactate concentration range of 2–30 mM, while the “eraser” enzyme SIRT2 is responsible for catalyzing the delactylation of α-tubulin.^17^ Lactylation affects protein structure and function by neutralizing the positive charge of lysine residues, thereby altering their spatial conformation, functional activity, subcellular localization,^27^ and interactions with other molecules. Notably, when lactylation occurs on histones at gene promoter regions, it influences gene transcription by modulating chromatin accessibility. To date, three lactylation “reader” proteins have been identified that specifically recognize histone lactylation marks: Brg1^28^ and TRIM33^29^ as main readers of H3K18la, and DPF2^30^ as a reader of H3K14la, all of which promote downstream gene transcription (Fig. 1).

### Crosstalk between lactylation and other PTMs

Due to the chemical reactivity of ε-amino groups, lysine residues serve as modification sites for various PTMs, including acetylation, methylation, and ubiquitination.^31^ Consequently, lactylation competes with these PTMs for shared lysine sites, leading to crosstalk that influences protein stability,^32^^,^^33^ molecular interactions, and gene transcription. Notably, no lactylation-specific acyltransferases have been identified to date. Instead, lactylation-modifying enzymes typically participate in regulating various acylation modifications, such as acetylation, succinylation, crotonylation, 2-hydroxyisobutyrylation, and β-hydroxybutyrylation.^34^ Studies indicate that the binding efficiency of acyltransferases correlates with substrate chain length, explaining why enzymes like p300 generally exhibit higher affinity for acetyl-CoA than for lactyl-CoA. Quantitative analyses of cultured cells and mouse cardiac tissues revealed that lactyl-CoA concentrations are 20- to 350-fold lower than those of major acyl-CoAs (e.g., acetyl-CoA, propionyl-CoA, and succinyl-CoA).^35^ These findings highlight the need to elucidate the precise mechanisms by which acyltransferases catalyze lactylation under such conditions. Notably, research in glioblastoma demonstrates that CBX3 can bind p300 and promote its preferential use of lactyl-CoA over acetyl-CoA to drive histone lactylation.^36^ Currently, the dynamic relationship between lactylation and other PTMs at the same site has been rarely reported, with a few studies suggesting that their crosstalk can occur through intermediary proteins, as exemplified by acetylation. The interaction between these two modifications can regulate protein activity, the assembly of protein complexes, and gene expression in a context-dependent manner. For instance, in hepatocellular carcinoma (HCC), p300-mediated acetylation of PDHX at Lys488 disrupts the assembly of the pyruvate dehydrogenase complex (PDC), promoting lactate production and subsequently inducing H3K56la.^37^ In ovarian cancer, lactate derived from glutaminolysis is enhanced via lysine 156 acetylation of ME2 by ACAT1, which increases ME2 enzymatic activity and indirectly facilitates lactylation-mediated homologous recombination repair.^38^ These studies reveal a bidirectional relationship in which acetylation modulates the activity of enzymes driving lactate production, while lactylation exerts downstream regulatory effects, forming a feedback loop that tightly links metabolic states to cellular functions.

### Strength of evidence levels in lactylation experiments

Before proceeding with the subsequent discussion, it is necessary to first understand the differences in the strength of evidence levels among various lactylation-related experiments. Observing changes in intracellular or extracellular lactate concentrations represents the lowest tier of evidence, as this approach does not necessarily reflect changes in lactylation levels. A more reliable method involves detecting changes in the lactylation levels of certain amino acid residues, which indicates a correlation between lactylation modifications and cellular phenotypes but cannot establish causality. Stronger evidence is provided by experiments where the addition of lactate dehydrogenase inhibitors (LDHis) or sodium lactate alters the lactylation levels at specific target sites and leads to corresponding changes in cellular phenotypes. However, this method might indirectly impact cellular outcomes by broadly affecting glycolytic pathways or other lactylation sites through the use of supra-physiological lactate concentrations. The highest level of evidence is achieved through site-directed mutagenesis to simulate delactylation^39^ or by mimicking lactylation modifications using orthogonal translation systems with pyrrolysyl-tRNA synthetases.^14^, ^15^, ^16^ The highest evidence classification (Level III–IV) for some lactylation-related mechanistic research is summarized in Supplementary Table 1.

## Lactylation enhances cellular adaptation and tumor innate resistance

### Lactylation in normal tissues and benign lesions

Although lactylation was discovered relatively recently, it is evolutionarily conserved and widely prevalent, similar to the Warburg effect. Numerous lactylation sites have been identified across diverse organisms, from single-celled species to multicellular eukaryotes, including normal tissues, benign lesions, and tumors. For instance, studies in *Escherichia coli* have identified 446 endogenous Kla sites regulated by the eraser enzyme CobB, along with 79 potential targets of the writer enzyme YiaC.^40^ In maize, 37 Kla sites on 16 histones have been reported, with lactylation being implicated in drought stress tolerance.^41^

Lactylation also occurs in normal animal and human tissues, where it is frequently up-regulated during biological processes involving glycolytic activation, such as spermatogenesis,^42^ preimplantation and early embryonic development,^43^, ^44^, ^45^ and neural and brain development.^17^^,^^46^^,^^47^ Notably, glycolysis, lactate, and lactylation are closely linked to cellular adaptation under environmental stress. For example, the marine microalga Nannochloropsis oceanica exhibits elevated lactylation under nitrogen deprivation.^48^ In spermatozoa, low glucose conditions induce lactylation of G6PD, enhancing its enzymatic activity to boost the pentose phosphate pathway, thereby improving antioxidant capacity and maintaining motility in glucose-deprived environments.^49^

**Figure 2:**
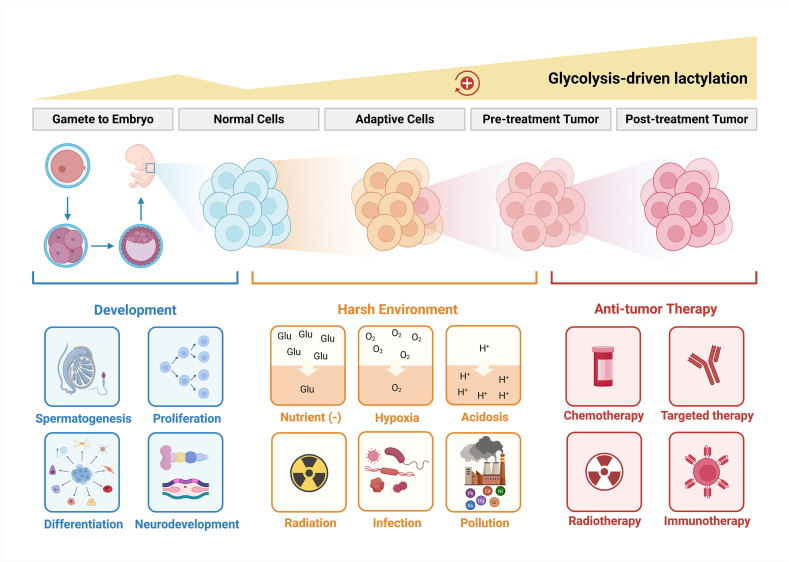
Dynamics of lactylation modifications across physiological and pathological states. Lactylation plays pivotal regulatory roles in both physiological processes and pathological conditions such as cancer. The intracellular lactylation levels are often up-regulated under the following contexts: (1) from gametogenesis through embryonic development until organogenesis completion; (2) when normal tissues undergo adaptive metabolic reprogramming in response to hostile microenvironments; and (3) during tumorigenesis progression and therapy-induced development of chemoresistance in malignancies, with these observations primarily derived from cell lines, patient-derived organoids and patient tissues (see Table S1). Created with BioRender.com.

Moreover, lactylation plays a protective role in stress responses. After traumatic brain injury, neurons increase histone lactylation to up-regulate PSMD14, inhibiting PANoptosis.^50^ Lithocholic acid alleviates mycotoxin-induced intestinal epithelial inflammation and oxidative stress via PPARγ-mediated histone acetylation and lactylation.^51^ Additionally, up-regulated lactylation is critical for activating inflammatory responses against pathogen infection and xenobiotic toxins, as well as subsequent fibrotic repair. For instance, macrophages^52^ and CD8+ T cells^53^ exhibit enhanced glycolysis and lactylation during functional activation. Similarly, lung tissues exposed to environmental pollutants (e.g., PM2.5, silica dust, and arsenic) up-regulate glycolysis and lactylation to promote fibrogenesis.^54^, ^55^, ^56^ Lactylation also contributes to glial scar formation in neurological stress,^57^ as well as hepatic, renal,^58^ and cardiac fibrosis^59^ (Fig. 2).

### Association between lactylation and tumorigenesis

It is well-established that tumor cells originate from normal cells but exhibit significantly enhanced aerobic glycolysis and lactylation modifications, which has naturally drawn scientific attention to their potential roles in oncogenesis. The scientific community has proposed multiple theories regarding tumor origins, including: (1) mitochondrial respiratory dysfunction or mitochondria-nuclear communication abnormalities,^60^ (2) tissue field organization theory,^61^ and (3) phylogenetic system theory.^62^ Notably, the mainstream somatic mutation theory is increasingly being challenged. For instance, studies have demonstrated that various carcinogens do not directly damage DNA, suggesting that hotspot driver mutations may primarily result from endogenous processes.^63^ Furthermore, recent research in Drosophila revealed that transient, reversible depletion of Polycomb proteins can induce a persistent cancerous state without driver mutations.^64^ These findings compel us to reconsider the potential role of metabolism-coupled epigenetic reprogramming in promoting tumorigenesis when normal cells undergo environmental adaptation.

Recent studies have shown that adaptive pressures from hostile microenvironments can select for malignant cells with Warburg metabolic phenotypes. One research team subjected low-glycolysis breast ductal carcinoma *in situ* (DCIS) cells to prolonged (months-long) combined selective pressures mimicking the initial tumorigenic microenvironment, including hypoxia, acidosis, and nutrient deprivation. Single-cell clones isolated at the experimental endpoint demonstrated significantly increased proportions of cells exhibiting aerobic glycolysis phenotypes through metabolic and transcriptomic profiling. Remarkably, these cells maintained their Warburg metabolic characteristics even after multiple generations of culture under nutrient- and oxygen-replete conditions^65^ (Fig. 2).

### Positive feedback loops of lactylation in pathological states

Notably, numerous studies have revealed that lactylation modifications induced by activated Warburg metabolism under pathological conditions participate in various positive feedback mechanisms that maintain their aberrant up-regulation. Histone lactylation-mediated positive feedback loops can be categorized into three predominant patterns: (1) Histone lactylation enrichment at promoters of glycolytic enzymes (HK2, PKM2, and LDHA)^66^, ^67^, ^68^; (2) Histone lactylation-mediated suppression of the expression of the lactylation eraser enzymes HDAC2/3^69^^,^^70^; and (3) Lactylation-promoted transcriptional activation of downstream proteins that enhance glycolysis through multiple mechanisms, including up-regulating transcription of aerobic glycolytic enzymes,^71^, ^72^, ^73^, ^74^ increasing mRNA methylation to boost expression,^75^ altering ubiquitination to improve stability,^76^ or modifying phosphorylation states to augment enzymatic activity^77^ (Fig. 5).

**Figure 3:**
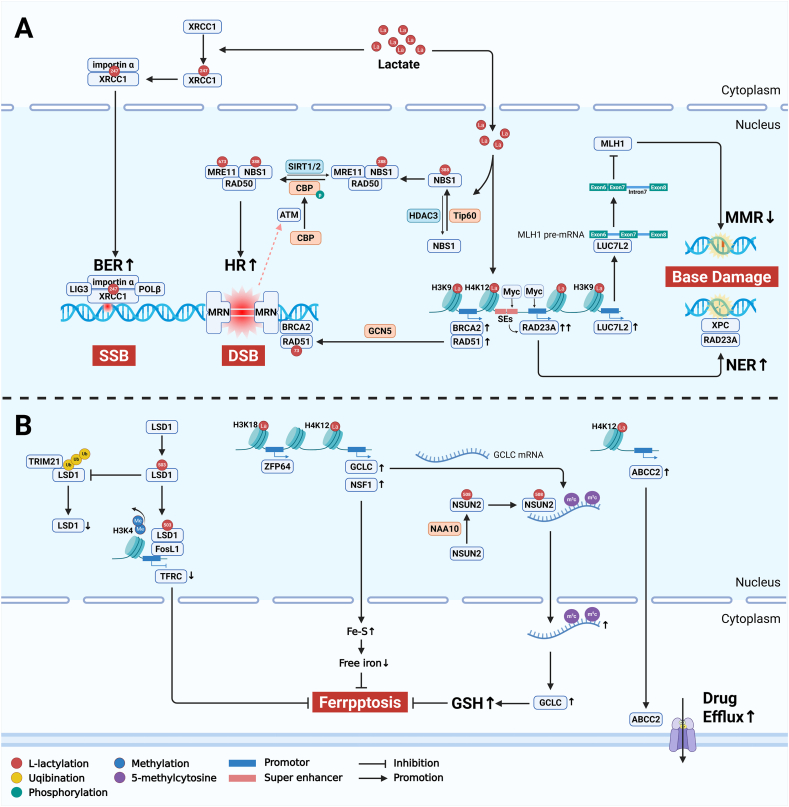
Lactylation-mediated mechanisms of tumor radio/chemoresistance. **(A)** Chemoradiotherapy can eliminate tumor cells by inducing DNA base damage, single-strand breaks, and double-strand breaks; however, tumor cells may develop resistance through histone lactylation and non-histone lactylation modifications, which up-regulate base excision repair, nucleotide excision repair, and homologous recombination repair. In radiotherapy-resistant glioblastoma cell lines, lactylation-mediated alternative splicing suppresses the mismatch repair pathway, thereby conferring temozolomide treatment resistance. **(B)** Additionally, lactylation-dependent regulation of glutathione and iron ions to inhibit ferroptosis, along with the up-regulation of ABC transporters, also plays a pivotal role in tumor resistance to chemoradiotherapy. La lactate. Created with BioRender.com.

**Figure 4:**
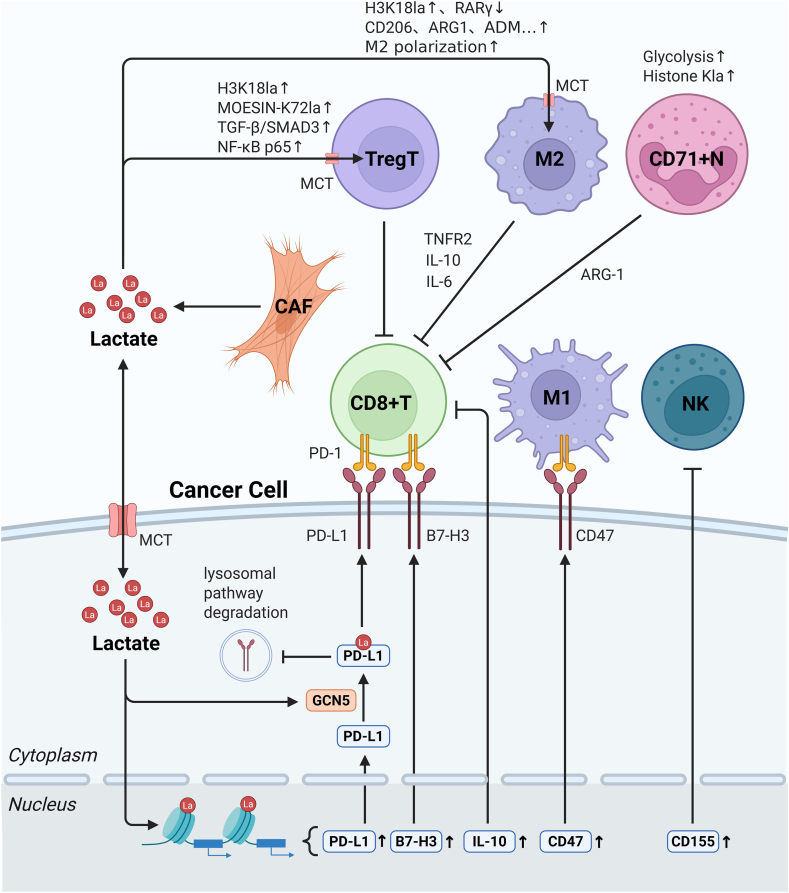
Mechanism of lactylation-mediated immunotherapeutic resistance. The up-regulated aerobic glycolysis in tumor cells or cancer-associated fibroblasts generates substantial lactate. Within tumor cells, elevated histone lactylation promotes the transcription of immunosuppressive molecules, thereby impairing the tumor-killing capacity of immune cells such as CD8+ T cells. Additionally, lactate in the tumor microenvironment can indirectly contribute to immunotherapeutic resistance by up-regulating lactylation modifications, which facilitates the recruitment of regulatory T cells (Tregs) and induces M2 polarization of macrophages. La lactate. Created with BioRender.com.

**Figure 5:**
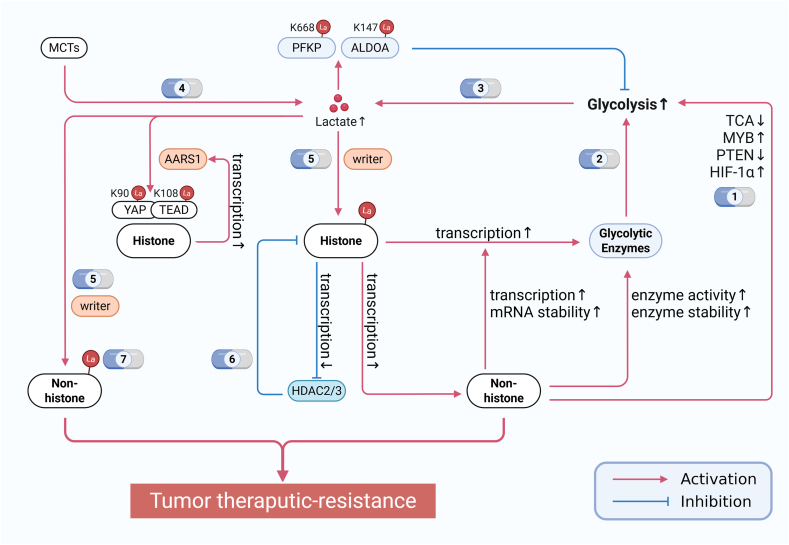
Feedback regulation of lactylation and targeting strategies. Lactylation modifications exhibit both positive and negative feedback mechanisms. The positive feedback mechanism primarily relies on histone or non-histone lactylation to directly or indirectly regulate the expression levels of glycolytic enzymes. Currently, targeting different nodes of lactylation modifications, particularly those that disrupt the positive feedback loop, holds significant therapeutic potential for cancer treatment. The number of therapeutic drug strategies in the figure corresponds to the first column in Table 1. La lactate. Created with BioRender.com.

In contrast, fewer studies have reported non-histone lactylation-involved positive feedback mechanisms. AARS1 was found to sense intracellular lactate and translocate into the nucleus to catalyze YAP-K90la/TEAD-K108la modifications. The lactylated complex resists ubiquitin-mediated degradation, forming a positive feedback loop through AARS1 transcriptional activation that promotes gastric cancer progression.^16^ (Fig. 5) Another study demonstrated that NUSAP1 forms a transcriptional regulatory complex with c-Myc and HIF-1α at the LDHA promoter region to increase its expression, while elevated lactate induces NUSAP1-K34la to prevent its own degradation, thereby establishing another positive feedback circuit.^78^

Interestingly, although multiple glycolytic enzymes in humans contain identified lactylation sites, only PFKP-K688la and ALDOA-K147la have been verified to inhibit their enzymatic activities and participate in glycolytic negative feedback.^79^^,^^80^ The functional significance of most non-histone protein lactylation sites remains largely unexplored, representing a substantial knowledge gap in this field (Fig. 5).

### Lactylation-mediated innate therapeutic resistance in tumors

The aforementioned evidence suggests that Warburg effect activation and aberrant lactylation modifications induced by hostile microenvironments likely represent key mechanisms through which precancerous or benign tumor cells acquire innate therapeutic resistance during malignant transformation. This connection becomes evident when considering the parallels between tumorigenic stressors and various cancer treatment modalities.

Hypoxia adaptation confers resistance to anti-angiogenic therapies. As reported in lung cancer and other malignancies, HIF-1α activation under hypoxia upregulates glycolysis, generating substantial l-lactate that subsequently promotes Kla modifications to facilitate hypoxic adaptation.^12^ Surprisingly, increased oxygen stimulation has also been reported to promote glioma growth and chemoresistance, suggesting that the positive feedback loops initiated by hypoxia-associated alterations either possess robust, sustained effects or induce diverse cellular plasticity.^81^ Furthermore, nutrient deprivation adaptation may enable metabolic flexibility to counteract oxidative stress from metabolic therapies or chemo/radiotherapy. For instance, pancreatic adenocarcinoma cells under glucose deprivation maintain mitochondrial respiration primarily through GLS1-mediated glutaminolysis, while NMNAT1 lactylation preserves nuclear NAD + salvage pathways to promote survival.^82^

Acidic microenvironment adaptation involves SLC4A4 up-regulation to maintain intracellular alkalinity, which enhances glycolysis-driven lactate accumulation. This process facilitates p53 lactylation-mediated death signal evasion and therapeutic resistance.^83^ Furthermore, the acidic tumor microenvironment (pH 6.5–7.1) generated by the Warburg effect can ionize weakly basic chemotherapeutic agents (anthracyclines, anthracenediones, and alkaloids) and even weakly acidic drugs like cisplatin through ion trapping, reducing their membrane permeability.^84^ The hypoxic, acidic niche also serves as a major immunosuppressive factor contributing to innate immune evasion and immunotherapeutic resistance, with detailed mechanisms discussed later.

Most significantly, the Warburg effect promotes broad-spectrum therapeutic resistance through lactylation-mediated cancer stemness enhancement and epithelial–mesenchymal transition. Pre-lactylation studies established tumor stemness as a pivotal resistance mechanism (see comprehensive reviews^85^^,^^86^). Recent evidence increasingly connects lactylation to therapeutic resistance: HCC and GBM tissues exhibit higher Kla levels than normal/adjacent tissues,^33^^,^^36^ with cancer stem cells showing elevated Kla compared to differentiated tumor cells. Lactylation critically maintains the stemness properties, e.g. PTBP1-K436la sustains glioma stemness.^33^ In HCC, histone lactylation enhances MCM7 expression, which reinforces stemness and radiotherapeutic resistance.^87^

## Mechanisms of lactylation-mediated therapeutic resistance in tumors

### Radiotherapy and chemotherapy

#### Promotion of DNA damage repair

Chemotherapeutic alkylating agents such as temozolomide primarily induce tumor cell apoptosis through base damage.^88^ Glioblastoma cells develop chemoresistance via XRCC1-K247la-mediated enhancement of base excision repair (BER). The lactylated XRCC1 exhibits a stronger affinity for the nuclear transport protein importin α, leading to increased nuclear translocation. Within the nucleus, XRCC1 serves as a scaffold protein that recruits DNA polymerase β and ligase 1/3 to fill gaps and ligate ends, thereby completing short-patch repair of simple breaks.^39^ Temozolomide can also eliminate tumor cells through mismatch repair (MMR)-dependent futile repair triggered by alkylated bases. Studies have reported that glioblastoma H3K9la upregulates LUC7L2 transcription, mediating MLH1 intron 7 retention and consequently suppressing the MMR pathway to promote temozolomide resistance.^89^ However, most studies on histone-related lactylation like cannot manipulate lactylation modifications at specific gene promoter regions of histones. Instead, they often rely on demonstrating correlated changes in histone lactylation levels by knocking down LDHA/B using shRNA. This approach cannot rule out the possibility that the observed effects are mediated by lactate’s metabolic influence, its role as a signaling molecule, or the indirect or combined contributions of other lactylation sites in driving drug resistance. Another critical DNA damage repair pathway, nucleotide excision repair (NER), is also regulated by lactylation. H4K12la facilitates the formation of the niraparib-specific super-enhancer (Nira-SE) and recruits MYC to activate the promoter region of RAD23A, thereby enhancing DNA damage repair capacity in ovarian cancer cells.^90^ (Fig. 3A) When unrepaired base damage persists prior to DNA replication, or when high-energy radiation directly targets DNA or when therapy-induced ROS accumulation becomes excessive, severe DNA double strand breaks occur. Homologous recombination (HR) and non-homologous end joining (NHEJ) represent key mechanisms for cancer cells to counteract such damage. Current evidence primarily implicates Kla in promoting tumor DNA repair through HR-associated MRN complexes. The MRN complex, consisting of two MRE11, two NBS1, and two RAD50 proteins,^91^ senses DNA double-strand break sites and recruits ATM. Lactylation sites promoting HR have been identified on both MRE11 and NBS1. For instance, phosphorylated CBP catalyzes MRE11-K673la to enhance DNA binding and accelerate end resection.^92^ TIP60-mediated NBS1–K388la strengthens NBS1 interaction with MRE11-RAD50, facilitating MRN complex formation and HR protein recruitment to DSB sites.^93^ Additionally, GCN5 not only mediates H3K9la to directly up-regulate RAD51 and BRCA2 expression, enhancing HR, but also may potentiate cisplatin resistance through RAD51-K73la.^94^ Furthermore, bladder cancer H3K18la promotes YBX1 and YY1 expression, potentially up-regulating DNA damage repair genes to mediate cisplatin resistance.^95^ (Fig. 3A).

#### Suppression of ferroptosis

In addition to causing DNA damage, elevated ROS induced by radio/chemotherapy can trigger tumor cell ferroptosis through lipid peroxidation. Emerging evidence has demonstrated that tumor cells develop acquired resistance by inhibiting ferroptosis via lactylation modifications. For instance, gastric cancer NSUN2–K508la promotes m5C modification and mRNA stabilization of GCLC, leading to increased intracellular glutathione levels that reduce lipid peroxidation and confer resistance to doxorubicin-induced ferroptosis.^23^ Colorectal cancer stem cells employ H4K12la to directly up-regulate GCLC transcription, thereby inhibiting ferroptosis and developing oxaliplatin resistance.^96^ Cancer-associated fibroblast (CAF)-derived lactate promotes H3K18la-mediated ZFP64 expression in triple-negative breast cancer (TNBC) cells, suppressing ferroptosis and inducing doxorubicin resistance.^97^ (Fig. 3B) In lung cancer cells, elevated histone lactylation at the AIM2 promoter enhances its expression. The up-regulated AIM2 suppresses STAT5b phosphorylation and promotes proteasomal degradation, ultimately attenuating ACSL4-mediated ferroptosis.^98^

Lactylation-induced ferroptosis suppression may also mediate therapeutic resistance to other treatment modalities. In BRAFi/MEKi-resistant melanoma cells, LSD1-K503la facilitates its interaction with FosL1, preventing TRIM21-mediated ubiquitination and degradation. This process cooperatively down-regulates TFRC transcription to reduce iron uptake and inhibit ferroptosis.^99^ Hepatocellular carcinoma H3K18la enhances NFS1 transcriptional expression, reducing susceptibility to ferroptosis following microwave ablation.^100^

#### Enhancement of drug efflux transport

The increase of drug efflux represents one of the pivotal mechanisms underlying chemoresistance in tumor cells. Although current reports on lactylation-mediated regulation of drug efflux proteins remain limited, emerging evidence suggests its potential involvement. In dormant-like colorectal cancer cells, reduced SMC4 expression up-regulates glycolytic enzymes, leading to increased lactate production. This metabolic shift drives H4K12la enrichment at the promoter regions of ABC transporter genes, resulting in their transcriptional activation and subsequent development of irinotecan resistance. Notably, this resistance phenotype can be reversed by co-treatment with the ABCC2 inhibitor propionic acid, demonstrating the functional significance of this lactylation-dependent efflux mechanism^101^ (Fig. 3B).

### Targeted therapy

#### Angiogenesis-targeted therapy

The functional peptide P4-135aa encoded by MAPK6P4 phosphorylates KLF15 at S238, enhancing KLF15 protein stability and nuclear translocation to promote LDHA transcription. LDHA subsequently binds and facilitates lactylation modifications of VEGFR2 and VE-cadherin, upregulating their expression and ultimately promoting vascular mimicry in glioblastoma (GBM).^102^ Lactylation-mediated stabilization of HIF1α serves as a transcriptional enhancer for KIAA1199, whose overexpression promotes angiogenesis and vascular mimicry formation through increased secretion of VEGFA, thereby mediating resistance to anti-angiogenic therapy.^103^ In HCC, GP73 is activated through histone lactylation and c-Myc, which directly binds to STAT3 and enhances GRP78-induced endoplasmic reticulum stress, stimulating downstream STAT3 phosphorylation. This STAT3 activation potentiates GP73-mediated pro-angiogenic functions, conferring resistance to anti-angiogenic therapy.^104^

H3K18la promotes the transcription of RUBCNL/Pacer, which interacts with BECN1 to mediate the recruitment and functional activation of class III phosphatidylinositol 3-kinase complexes, thereby facilitating autophagosome maturation. This process enhances colorectal cancer cell survival under hypoxic conditions and confers resistance to bevacizumab treatment.^105^

In advanced HCC, enhanced glycolysis leads to lactate accumulation and IGF2BP3 lysine lactylation. This lactylation modification is crucial for capturing PCK2 and NRF2 mRNAs, thereby augmenting their expression. The process reprograms serine metabolism and strengthens antioxidant defense systems. Furthermore, altered serine metabolism increases the availability of the methylation substrate SAM for m6A methylation of the PCK2 and NRF2 mRNAs. The lactylated IGF2BP3-PCK2-SAM-m6A cycle sustains elevated PCK2 and NRF2 levels, reinforcing antioxidant systems and promoting lenvatinib resistance in HCC.^106^ Additional studies revealed that H3K18la enhances HECTD2 transcription, which functions as an E3 ubiquitin ligase for KEAP1 to promote its proteasomal degradation, consequently activating the KEAP1/NRF2 signaling pathway to initiate antioxidant responses and mediate lenvatinib resistance.^107^

#### Other targeted therapies

Non-small cell lung cancer (NSCLC) develops EGFR-TKI resistance through dual positive feedback loops involving EGR1/NNMT/EGR1 and NNMT/ALDH3A1/H3K18la/NNMT.^108^ In the tumor microenvironment, CTHRC1-positive CAFs secrete CTHRC1, which binds to TGFBR2 via its C-terminal domain, recruiting TGF-β1 to activate the TGF-β/Smad3 signaling cascade. This pathway up-regulates HK2, enhancing glycolytic activity in cancer cells and promoting excessive lactate secretion. The accumulated lactate further increases CTHRC1 expression in CAFs through H3K18la modification, establishing a self-sustaining positive feedback loop that maintains EGFR-TKI resistance.^73^

In prostate cancer, prolonged enzalutamide (Enz) treatment induces SLC4A4 up-regulation, which mediates P53 lactylation through the NF-κB/STAT3/SLC4A4 axis, ultimately leading to Enz resistance.^83^ Multiple myeloma patients develop proteasome inhibitor resistance through an alternative mechanism involving extrachromosomal circular DNA (eccDNA). The amplified eccDNA increases KIF3C expression while reducing MUC20 levels, thereby alleviating MUC20-mediated suppression of IGF-1R lactylation. The consequent up-regulation of MET signaling confers resistance to proteasome inhibitors.^109^

### Immunotherapy

#### Suppression of anti-tumor immune cells

Multiple malignancies, including non-small cell lung cancer (NSCLC), hepatocellular carcinoma (HCC), acute myeloid leukemia (AML), and head and neck squamous cell carcinoma (HNSCC), exploit elevated glycolysis or high-lactate microenvironments to up-regulate histone lactylation (primarily H3K18la) at immune checkpoint gene promoters. This epigenetic modification enhances the transcriptional expression of immunosuppressive molecules such as PD-L1,^24^^,^^110^, ^111^, ^112^, ^113^ B7–H3,^114^ CD47,^36^ CD155,^115^ and IL-10.^116^ Notably, PD-L1 itself undergoes lactylation modification that prevents its lysosomal degradation, thereby stabilizing protein expression levels.^112^ These immunosuppressive factors collectively create a tumor microenvironment that potently inhibits CD8+ T cell-mediated anti-tumor immunity, facilitating immune evasion (Fig. 4).

Emerging evidence reveals that lactate derived from KRAS-mutant tumor cells can induce histone lactylation in tumor-specific cytotoxic T lymphocytes (CTLs), directly activating circATXN7 transcription. This process renders CTLs more susceptible to activation-induced cell death (AICD) through an NF-κB/p65 subunit-dependent mechanism.^117^

The tumor microenvironment further recruits bystander immune cells to reinforce immunosuppression. In glioblastoma, the tumor-derived PERK-ATF4 axis activates monocyte-derived macrophages (MDMs), up-regulating GLUT1 expression and enhancing glycolysis. The resulting lactate accumulation drives histone lactylation in MDMs, promoting IL-10 expression.^118^ Remarkably, even without direct tumor cell involvement, CD71+ neutrophils in glioblastoma hypoxic niches can autonomously up-regulate glycolysis, induce self-histone lactylation, and increase ARG1 transcription.^119^ These findings position IL-10 and ARG1 as key mediators of myeloid-derived immunosuppression (Fig. 4).

#### Recruitment and potentiation of immunosuppressive cells

Lactate in the tumor microenvironment (TME) promotes regulatory T cell (Treg) recruitment and enhances their immunosuppressive functions through multiple pathways, thereby mediating immunotherapeutic resistance. For instance, in NSCLC, lactate enhances APOC2-K70la modification, increasing its stability. This promotes lipolysis and the subsequent release of extracellular free fatty acids, facilitating Treg recruitment.^120^ In anaplastic thyroid carcinoma, VSIG4 activates the PI3K/Akt-STAT3 pathway in tumor-associated macrophages (TAMs), driving glycolysis-mediated H3K18la up-regulation of the immunosuppressive factor SPP1 to recruit neutrophils and Tregs.^121^

Moreover, lactate in malignant pleural effusion (MPE) can be actively taken up by Tregs. MOESIN-K72la up-regulation strengthens the interaction between MOESIN and TGF-β receptor I, activating downstream SMAD3 signaling to amplify Treg generation and enhance their CD8+ T cell suppression.^122^ Additionally, H3K18la in Tregs regulates NF-κB p65 transcription, up-regulating TNFR2 and multiple immunosuppressive molecules.^123^ Beyond Tregs, lactate accumulation in the TME significantly induces METTL3 up-regulation in tumor-infiltrating myeloid cells (TIMs) via H3K18la. This mediates m6A modification of JAK1 mRNA, where the m6A-YTHDF1 axis enhances JAK1 translation efficiency and subsequent STAT3 phosphorylation, augmenting TIM immunosuppression.^124^

Lactate also orchestrates TAM-mediated immunosuppression. In pancreatic cancer, lactate-induced ENSA-K63la triggers STAT3-CCL2 signaling, increasing CCL2 secretion to recruit TAMs into the TME, thereby fostering an immunosuppressive niche.^125^ Long-term PI3K inhibitor (PI3Ki) treatment in prostate cancer feedback-activates the Wnt/β-catenin pathway, increasing nuclear β-catenin and compensatory lactate production. This reinduces H3K18la in TAMs, suppressing their phagocytic activity and driving therapeutic resistance.^126^ Furthermore, lactate-driven H3K18la in TAMs represses RARγ transcription, elevating IL-6 levels to confer pro-tumorigenic functions.^127^ (Fig. 4).

M2 polarization is critical for TAM immunosuppression. H3K18la plays a pivotal role by activating the transcription of M2 markers (CD206, ARG1, IL10, and ADM).^74^ Similarly, hypoxia-induced lactate secretion in glioma cells is taken up by macrophages via MCT1, promoting TNFSF9 expression through H3K18la to drive M2 polarization.^128^ In pancreatic cancer, the CTCF/HNRNPU/p300 axis up-regulates IGF2BP2 via enhanced promoter H3K18la. Elevated IGF2BP2 stabilizes CSF1 and MYC mRNAs, concurrently promoting TAM M2 polarization and tumor proliferation.^129^ (Fig. 4).

## Strategies targeting lactylation to overcome tumor therapeutic resistance

As elucidated in the previous sections, lactylation plays a pivotal role in mediating tumor resistance to various therapies. In most documented cases, tumors develop resistance to radiotherapy, chemotherapy, targeted therapy, and immunotherapy through up-regulated histone or non-histone lactylation modifications. Consequently, the primary objective in developing novel therapeutic sensitization strategies focuses on reducing lactylation levels in tumors. Based on the distinct components of the lactylation pathway, these intervention strategies can be categorized into three main approaches: (1) targeting lactate donors, (2) modulating lactylation-modifying enzymes, and (3) disrupting lactylated proteins and their associated signaling pathways (Fig. 5 and Table 1).

**Table 1:** Strategies targeting lactylation to overcome tumor therapeutic resistance.

Mechanism	Drugs	Targets	Cancer^a^	Research status
①Inhibit glycolysis upstream	MRTX1133	KRAS-G12D	PDAC^130^	Preclinical
	LGK974	Wnt	PC^126^	Phase II, 2014-2017, NCT02278133, Serious adverse events
②Inhibit glycolytic enzymes	2-DG	HK	BCa,^95^ CRC,^105^ NSCLC,^110^ EC,^131^ HCC,^132^ iCCA,^133^ LUAD^134^	Phase I, 2004–2008, NCT00096707, Manageable toxicity but limited efficacy (66% disease progression)
	JX06	PDHK1	HCC^111^	Preclinical
	Fargesin	PKM2	NSCLC^135^	Preclinical
	D34-919	PKM2-ALDH1A3	GBM^39^	Preclinical
	Shikonin	PKM2	Lung cancer^98^	Preclinical
③Inhibit Lac metabolism	Oxamate	LDHA	ccRCC,^136^ EC,^131^ NSCLC,^110^ BCa,^95^ iCCA,^133^	Preclinical
	FX11	LDHA	LUAD^137^	Preclinical
	GSK2837808A	LDHA	HCC^122^^,^^138^	Preclinical
	Stiripentol	LDHA/B	GBM^89^	Preclinical
	Isosafrole	LDHA/B	GBM^119^	Preclinical
	GNE-140	LDHA/B	GBM^118^	Preclinical
④Inhibit Lac intake	7ACC2	MCT1	MPE^139^	Preclinical
	AZD3965	MCT1	MPE,^123^ GBM^118^	Phase Ⅰ, 2013–2022, NCT01791595, Manageable toxicity but limited efficacy (46.2% disease progression)
⑤Inhibit Kla writer	A485	p300/CBP	Glioma^25^	Preclinical
	β-alanine	AARS1	CESC^15^	Preclinical
	MB-3	GCN5	OV^94^	Preclinical
⑥Activate Kla eraser	Honokiol	SIRT3	HCC^140^	Preclinical
	Gambogic acid	SIRT1	PC^141^	Preclinical
	SRT2104	SIRT1	GBM,^33^ GC^142^	Preclinical
⑦Inhibit Kla protein	Vorinostat	HDAC1-K412la	CRC^143^	Preclinical
	Trichostatin A			
	CPP^b^	MRE11-K673la	BRCA,^92^ CRC^92^	Preclinical
	CPP	ENSA-K63la	PDAC^130^	Preclinical
	anti-APOC2K70la antibody	APOC2-K70la	NSCLC^120^	Preclinical
	Tubuloside A	ABCF1-K430la	HCC^27^	Preclinical
	SBDA^b^	RCC2-K124la	BRCA^144^	Preclinical

a PDAC, pancreatic ductal adenocarcinoma; PC, prostate cancer; EC, endometrial carcinoma; CRC, colorectal cancer; HCC, hepatocellular carcinoma; BCa, bladder cancer; NSCLC, non-small cell lung cancer; iCCA, intrahepatic cholangiocarcinoma; LUAD, lung adenocarcinoma; ccRCC, clear cell renal cell carcinoma; GBM, glioblastoma; MPE, malignant pleural effusion; CESC, cervical cancer; OV, ovarian cancer; GC, gastric cancer; BRCA, breast cancer.

b SBDA, 5,5′-((4,4′-sulfonylbis(benzoyl))bis(azanediyl)) diisophthalic acid. CPP, cell penetrating peptide.

### Reduce lactate donors

The level of lactylation is closely associated with glycolytic pathway activation and the intracellular lactate concentration. Therefore, lactylation can be attenuated by either inhibiting upstream glycolytic activation pathways or directly targeting key glycolytic enzymes to reduce endogenous lactate production. Additionally, in certain contexts, targeting monocarboxylate transporter 1 (MCT1) to block exogenous lactate uptake represents another viable strategy for lactylation reduction.

For upstream glycolytic pathway inhibition, combination therapy with LGK974 (targeting Wnt/β-catenin signaling) and PI3Ki (copanlisib)/MEKi (trametinib) demonstrated complete tumor control in a genetically engineered mouse model of aggressive variant prostate cancer (AVPC; PTEN^fl/fl^Trp53 ^fl/fl^) by suppressing H3K18la and fully activating TAMs.^145^ Regarding glycolytic enzyme targeting, preclinical studies most frequently employ 2-deoxy-d-glucose (2-DG) to inhibit hexokinase (HK), or oxamate/GSK2837808A to suppress lactate dehydrogenase A (LDHA) activity. These monotherapies already exhibit antitumor efficacy, with 2-DG having progressed to phase II clinical trials. Their combination with conventional therapies (radiotherapy/chemotherapy, targeted therapy, or immunotherapy) may further overcome treatment resistance and enhance therapeutic outcomes. Notably, stiripentol, an FDA-approved antiepileptic drug with LDHA inhibitory properties, was shown to reduce DNA repair efficiency via the suppression of NBS1–K388la, thereby overcoming chemoresistance in murine models.^93^ For exogenous lactate uptake intervention, AZD3965 (an MCT1 inhibitor) combined with anti-PD-1 treatment significantly prolonged survival in malignant pleural effusion mouse models.^123^^,^^146^

### Targeting lactylation-modifying enzymes

Beyond lactate availability, lactylation-modifying enzymes represent crucial therapeutic targets. Current strategies primarily focus on inhibiting lactyltransferase (writer) enzymes. A485-mediated p300/CBP inhibition enhances radiosensitivity in gliomas,^25^ while MB-3 targeting GCN5 ameliorates cisplatin resistance in ovarian cancer.^94^ Additionally, β-alanine competitively inhibits AARS1, suppressing tumorigenesis in animal models.^15^ For lactylerase (eraser) enzymes, compounds like honokiol (a SIRT3 inhibitor),^140^ gambogic acid,^141^ and SRT2104 (SIRT1 inhibitor)^142^ disrupt lactylation-positive feedback loops by reducing histone lactylation, achieving both tumoricidal and therapy-sensitizing effects.

### Targeting lactylated proteins

Given the central role of glycolysis in carbon metabolism and the promiscuous acyltransferase activity of lactylation-modifying enzymes, directly intervening with lactylated proteins, or even specific lactylation sites, may offer more precise therapeutic efficacy with fewer off-target effects. Researchers have employed small-molecule screening and cell-penetrating peptide (CPP) design to specifically inhibit lactylation sites for therapy sensitization. For instance, D34-919 effectively disrupts the ALDH1A3-PKM2 interaction, preventing the enhancement of ALDH1A3-mediated PKM2 tetramerization. Both *in vitro* and *in vivo* studies have demonstrated that D34-919 treatment potentiates radio/chemotherapy-induced apoptosis in GBM cells.^39^ For other lactylation sites (ENSA-K63la, APOC2-K70la, and RCC2-K124la), designed CPPs or polyclonal antibodies enable specific blockade, achieving immunotherapy sensitization.^120^^,^^130^^,^^144^

## Challenges in targeting lactylation

Despite the numerous preclinical strategies summarized previously, their clinical translation presents significant hurdles. First, systemic inhibition of glycolysis or its upstream pathways (e.g., AZD3965) to reduce global lactylation risks induce adverse effects like gastrointestinal disorders or mediastinal disorders,^147^ a concern compounded by the fact that the activation of some antitumor immunity is also glycolysis- and lactylation-dependent.^53^ Furthermore, lactylation-modifying enzymes often exhibit pleiotropic functions, regulating other PTMs such as acetylation in normal cells, thereby posing a substantial risk of off-target effects. To mitigate these challenges pending the discovery of highly specific enzymes or cofactors, potential strategies include targeting the tumor microenvironment (TME) with drug carriers like lactate- or pH-responsive hydrogels and nanoparticles,^148^ or employing spatially-restricted gene editing via focused ultrasound.^149^ However, a more direct approach involves blocking functional lactylation sites with specific small molecules or peptides. Nevertheless, the efficacy of this strategy is questionable. Given the extensive network of lactylation-mediated resistance mechanisms, particularly histone lactylation, which orchestrates the transcription of numerous resistance genes, inhibiting a single site may prove insufficient to fully overcome therapeutic resistance. Moreover, this approach necessitates costly high-throughput PTM proteomics and subsequent drug screening, creating a significant bottleneck for its clinical application.

Perhaps the most profound challenge stems from the observation that not all therapy-resistant cells rely on enhanced glycolysis and lactylation, implying that targeted strategies may lack universal efficacy. In cisplatin-resistant ovarian cancer, for instance, while many models up-regulate glycolysis to drive chemoresistance,^150^^,^^151^ others exhibit a metabolic switch to fatty acid β-oxidation.^152^ Intriguingly, certain models with diminished GLUT1 expression and glucose uptake sustain lactate production and MRN complex lactylation via glutaminolysis.^38^ Further complicating the therapeutic landscape, some evidence suggests that lactylation can paradoxically exert anti-tumor effects. For example, METTL16 lactylation has been shown to induce cuproptosis in gastric cancer by promoting FDX1 expression and subsequent DLAT lipoylation. This apparent contradiction likely reflects the dynamic metabolic states of tumor cells. Some may shunt glucose from glycolysis into the pentose phosphate pathway to generate NADPH for counteracting oxidative stress, while others, like quiescent breast cancer cells, can enhance oxidative phosphorylation (OXPHOS) to acquire doxorubicin resistance.^153^ Adding another layer of complexity, reports indicate that decreased lactylation on proteins like METTL3 can actually enhance DNA damage repair in drug-tolerant persister (DTP) cells.^154^ Collectively, these contradictory findings underscore the profound heterogeneity of tumor cell dependence on lactylation, warranting more in-depth mechanistic investigations to delineate its context-specific roles.

## Conclusions and perspectives

Protein lactylation has been identified as an important PTM that directly links the Warburg effect to epigenetic regulation and gene expression. This review highlights that both histone and non-histone lactylation regulate diverse therapeutic resistance mechanisms, making this modification a potential contributor to treatment failure and disease progression in oncology.

However, targeting lactylation therapeutically remains highly complex. This complexity first arises from its sheer scale and diversity. Lactylation affects various histone and non-histone proteins, and individual proteins may carry multiple lactylation sites, which might have distinct or synergistic roles. This necessitates meticulous, site-specific functional validation, moving far beyond global measurements. Compounding this challenge is the dynamic and reversible nature of lactylation. Its regulation is exquisitely context-dependent, varying with cell type, metabolic state, and microenvironmental cues. Moreover, lactylation interacts with other PTMs, such as acetylation and ubiquitination, forming a regulatory network where its overall biological effect is challenging to predict and requires integrated analysis.

Despite these complexities, understanding the roles of lactylation remains a pressing priority. As a key downstream effector of the Warburg effect—a nearly universal hallmark of cancer—lactylation provides a direct mechanistic window into the metabolic underpinnings of tumorigenesis. Investigating its mechanisms is therefore not merely an academic exercise; it is essential for understanding the metabolic basis of cancer and may help identify new molecular therapies. Targeting this fundamental driver of malignancy represents a significant opportunity for major clinical translation, potentially benefiting a wide range of patients.

Therefore, future research may urgently address these critical challenges. In the realm of mechanistic exploration, key priorities should include: (1) Identification of novel lactylation-modifying enzymes: Identifying additional potential lactylation writers and erasers, and elucidating whether they exhibit target protein specificity. (2) Elucidating context-dependent regulation: Investigating how the subcellular compartmentalization of lactylation-modifying enzymes, the local metabolic microenvironment, and interacting proteins dynamically regulate lactylation preference, for instance, through allosteric regulation. (3) Mapping crosstalk networks: Employing multi-omics integration to dissect how lactylation engages in crosstalk with other PTMs through a coupled gene–metabolism–epigenome network. (4) Developing advanced manipulation tools: Creating more accessible and robust technologies for site-specific manipulation of lactylation to rigorously establish a causal link between lactylation and cellular functions.

For clinical translation, pivotal research directions include: (1) Optimizing combination therapies: actively exploring the safe therapeutic windows for inhibitors of glycolysis and lactate metabolism when combined with other treatments, aiming to maximize therapeutic enhancement while minimizing toxicity. (2) Patient stratification and biomarker discovery: Developing prognostic stratification for patients receiving lactylation-targeted therapies and identifying biomarkers to guide precision treatment. (3) Integrating biomedical engineering strategies: Proactively incorporating novel biomedical materials and non-invasive regulatory technologies into lactylation-targeting interventions to reduce side effects and improve therapeutic efficacy.

In conclusion, while the complexity of lactylation presents significant research challenges, it also offers promising opportunities for therapeutic development. Concerted, multi-disciplinary efforts to navigate this complexity are essential to unlock a new generation of metabolism-targeted precision therapies in oncology.

## CRediT authorship contribution statement

**Wanghao Zhang:** Writing – original draft, Visualization, Investigation, Data curation. **Guanglong Huang:** Funding acquisition, Data curation. **Woheng Tang:** Investigation, Data curation. **Jiaxian Li:** Data curation. **Jingxian Chen:** Data curation. **Yaojiang Feng:** Data curation. **Kaichen Li:** Data curation. **Can Pan:** Data curation. **Shunshen Li:** Data curation. **Huayang Zhang:** Data curation. **Rongxu Ye:** Data curation. **Hao Long:** Writing – review & editing, Supervision. **Guo-zhong Yi:** Writing – review & editing, Supervision, Funding acquisition, Data curation.

## Generative AI statement

The authors declare that no Generative AI was used in the creation of this manuscript.

## Funding

This study was supported by the 10.13039/501100001809National Natural Science Foundation of China (No. 82272636, 82473161) and the Guangdong Science and Technology Department (China) (No. 2024A1515011749, 2023A1515012382). The funder had no role in the study design, data collection and analysis, decision to publish, or preparation of the manuscript.

## Conflict of interests

The authors declare no conflict of interests.
